# *N*-[Morpholino(phen­yl)meth­yl]benzamide

**DOI:** 10.1107/S1600536809005327

**Published:** 2009-02-21

**Authors:** L. Muruganandam, S. Rajeswari, D. Tamilvendan, V. Ramkumar, G. Venkatesa Prabhu

**Affiliations:** a Department of Chemistry, National Institute of Technology, Trichy 620 015, India; b Department of Chemistry, Indian Institute of Technology–Madras, Chennai 600 036, India

## Abstract

The title compound, C_18_H_20_N_2_O_2_, crystallizes with two mol­ecules in the asymmetric unit. The morpholine rings of both mol­ecules adopt chair conformations. The crystal structure is stabilized by inter­molecular N—H⋯O hydrogen bonds. One phenyl ring is disordered over two orientations in a 0.665 (5):0.335 (5) ratio.

## Related literature

For background literature on benzamides and morpholines, see: Carbonnelle *et al.* (2005 ▶); Hatzelmann & Schudt (2001 ▶); Li *et al.* (1998 ▶); Malik *et al.* (2006 ▶); Sedavkina *et al.* (1984 ▶); Simonini *et al.* (2006 ▶); Suzuki *et al.* (2005 ▶); Zhou *et al. *(1999); Zhou *et al.* (1999 ▶). For ring conformations, see: Cremer & Pople (1975 ▶).
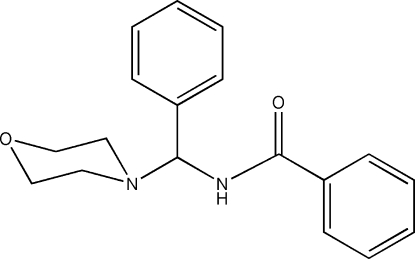


## Experimental

### 

#### Crystal data

C_18_H_20_N_2_O_2_
*M* *_r_* = 296.36
Triclinic, 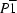

*a* = 9.9190 (3) Å
*b* = 10.6793 (3) Å
*c* = 15.8050 (5) Å
α = 79.747 (2)°
β = 85.543 (1)°
γ = 85.467 (1)°
*V* = 1638.84 (9) Å^3^
*Z* = 4
Mo *K*α radiation
μ = 0.08 mm^−1^
*T* = 295 K
0.20 × 0.19 × 0.08 mm

#### Data collection

Bruker APEXII CCD diffractometer
Absorption correction: multi-scan (*SADABS*; Bruker, 1999 ▶) *T*_min_ = 0.979, *T*_max_ = 0.994
21213 measured reflections
7329 independent reflections
4069 reflections with *I* > 2σ(*I*)
*R*_int_ = 0.033

#### Refinement

*R*[*F* ^2^ > 2σ(*F* ^2^)] = 0.052
*wR*(*F* ^2^) = 0.156
*S* = 1.03
7329 reflections
417 parameters
1 restraint
H atoms treated by a mixture of independent and constrained refinement
Δρ_max_ = 0.24 e Å^−3^
Δρ_min_ = −0.19 e Å^−3^

### 

Data collection: *APEX2* (Bruker, 2004 ▶); cell refinement: *SAINT* (Bruker, 2004 ▶); data reduction: *SAINT*; program(s) used to solve structure: *SHELXS97* (Sheldrick, 2008 ▶); program(s) used to refine structure: *SHELXL97* (Sheldrick, 2008 ▶); molecular graphics: *ORTEP-3* (Farrugia, 1997 ▶); software used to prepare material for publication: *SHELXL97*.

## Supplementary Material

Crystal structure: contains datablocks global, I. DOI: 10.1107/S1600536809005327/hb2906sup1.cif

Structure factors: contains datablocks I. DOI: 10.1107/S1600536809005327/hb2906Isup2.hkl

Additional supplementary materials:  crystallographic information; 3D view; checkCIF report

## Figures and Tables

**Table 1:** Hydrogen-bond geometry (Å, °)

*D*—H⋯*A*	*D*—H	H⋯*A*	*D*⋯*A*	*D*—H⋯*A*
N4—H4⋯O2	0.85 (2)	2.11 (2)	2.932 (2)	163.5 (17)
N2—H2⋯O4^i^	0.832 (19)	2.10 (2)	2.918 (2)	166.5 (17)

Symmetry code: (i) 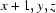.

**Fig. 1.**
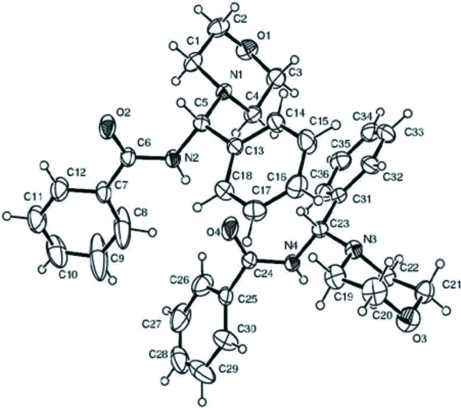
The molecular structure of (I); displacement ellipsoids for the non-hydrogen atoms are drawn with 30% probability.

## supplementary crystallographic information

### Comment

Benzamide derivatives, known for their anti-inflammatory and immunomodulatory (Hatezelmann *et al.*, 2001; Carbonnelle *et al.*, 2005), anti-tumoral (Suzuki *et al.*, 2005), antipsychotic (Simonini *et al.*, 2006), and antiallergic (Zhou *et al.*,1999) activities, are drugs widely used in medicine (Malik *et al.*, 2006).

Morpholine is a multipurpose chemical which is used as a solvent for resins, dyes and waxes. One of its most important use is as a chemical intermediate in the preparation of pesticides (Li *et al.*, 1998). A number of morpholine derivatives have been described as analgesics and local anesthetics. The morpholinomethyl derivative of pyrizinamide (morphozinamide) has been found to be more effective in the treatment of tuberculosis than pyrizinamide (Sedavkina *et al.*, 1984).

In the title compound, (I), each of the two independent molecules contains three ring systems, one phenyl ring, one benzamide and a morpholino ring (Fig. 1).

The morpholine rings of the two molecules adopts the usual chair conformation (Q_T_ = 0.577 (2) Å, q(2)=0.012 (2)Å and q(3)=0.577 (2) Å, θ = 1.4 (2)°. (Cremer & Pople, 1975). The best planes of the rings pass through the C atoms, leaving the O and N atoms on either side. The methine (C—H) substitution (C5) of the morpholine ring is in an equatorial position. One of the benzamide moiety in the crystal structure is disordered with an occupancy factor of 0.33 and 0.67. The phenyl moiety is planar. The crystal structure is stabilized by intermolecular N—H···O hydrogen bonds (Table 1).

### Experimental

Benzamide (12.1 g, 0.1 mol) was dissolved in a minimum quantity of ethanol. To this solution, benzaldehdye (10 ml, 0.1 mol) followed by morpholine (9 ml, 0.1 mol) was added in small quantities with constant stirring in an ice bath. For about 2 hrs, the mixture was kept at ice cold temperature. After 10 days a pale yellow semi-solid was obtained. The product was purified by washing with distilled water several times and finally with 5 ml of acetone. The compound was dried in an air oven at 80° C and recrystallized from ethanol to yield colourless slabs of (I).

### Refinement

One of the benzamide groups in the crystal structure is disordered with occupancy factor of 0.33 and 0.67. The H atoms were positioned geometrically and refined using riding model, with C—H = 0.93 Å and *U*_iso_(H) = 1.2*U*_eq_(C) for aromatic C—H, C—H = 0.97 Å and *U*_iso_(H) = 1.2*U*_eq_(C) for CH2. The H atoms associated with nitrogen atoms were located from difference maps and refined.

### Crystal data

**Table:**

C_18_H_20_N_2_O_2_	*Z* = 4
*M**_r_* = 296.36	*F*(000) = 632
Triclinic, *P*1	*D*_x_ = 1.201 Mg m^−^^3^
Hall symbol: -P 1	Mo *K*α radiation, λ = 0.71073 Å
*a* = 9.9190 (3) Å	Cell parameters from 4916 reflections
*b* = 10.6793 (3) Å	θ = 2.2–23.9°
*c* = 15.8050 (5) Å	µ = 0.08 mm^−^^1^
α = 79.747 (2)°	*T* = 295 K
β = 85.543 (1)°	Slab, colourless
γ = 85.467 (1)°	0.20 × 0.19 × 0.08 mm
*V* = 1638.84 (9) Å^3^	

### Data collection

**Table:**

Bruker APEXII CCD diffractometer	7329 independent reflections
Radiation source: fine-focus sealed tube	4069 reflections with *I* > 2σ(*I*)
graphite	*R*_int_ = 0.033
ω scans	θ_max_ = 28.1°, θ_min_ = 1.3°
Absorption correction: multi-scan (*SADABS*; Bruker, 1999)	*h* = −10→12
*T*_min_ = 0.979, *T*_max_ = 0.994	*k* = −14→14
21213 measured reflections	*l* = −20→18

### Refinement

**Table:**

Refinement on *F*^2^	Secondary atom site location: difference Fourier map
Least-squares matrix: full	Hydrogen site location: inferred from neighbouring sites
*R*[*F*^2^ > 2σ(*F*^2^)] = 0.052	H atoms treated by a mixture of independent and constrained refinement
*wR*(*F*^2^) = 0.156	*w* = 1/[σ^2^(*F*_o_^2^) + (0.0693*P*)^2^ + 0.2499*P*] where *P* = (*F*_o_^2^ + 2*F*_c_^2^)/3
*S* = 1.03	(Δ/σ)_max_ < 0.001
7329 reflections	Δρ_max_ = 0.24 e Å^−^^3^
417 parameters	Δρ_min_ = −0.19 e Å^−^^3^
1 restraint	Extinction correction: *SHELXL97* (Sheldrick, 2008), Fc^*^=kFc[1+0.001xFc^2^λ^3^/sin(2θ)]^-1/4^
Primary atom site location: structure-invariant direct methods	Extinction coefficient: 0.0061 (15)

### Special details

**Table:**

Geometry. All e.s.d.'s (except the e.s.d. in the dihedral angle between two l.s. planes) are estimated using the full covariance matrix. The cell e.s.d.'s are taken into account individually in the estimation of e.s.d.'s in distances, angles and torsion angles; correlations between e.s.d.'s in cell parameters are only used when they are defined by crystal symmetry. An approximate (isotropic) treatment of cell e.s.d.'s is used for estimating e.s.d.'s involving l.s. planes.
Refinement. Refinement of *F*^2^ against ALL reflections. The weighted *R*-factor *wR* and goodness of fit *S* are based on *F*^2^, conventional *R*-factors *R* are based on *F*, with *F* set to zero for negative *F*^2^. The threshold expression of *F*^2^ > σ(*F*^2^) is used only for calculating *R*-factors(gt) *etc*. and is not relevant to the choice of reflections for refinement. *R*-factors based on *F*^2^ are statistically about twice as large as those based on *F*, and *R*- factors based on ALL data will be even larger.

### Fractional atomic coordinates and isotropic or equivalent isotropic displacement parameters (Å^2^)

**Table:**

	*x*	*y*	*z*	*U*_iso_*/*U*_eq_	Occ. (<1)
H4	0.424 (2)	0.6985 (17)	0.1586 (12)	0.046 (5)*	
H2	0.920 (2)	0.7527 (17)	0.1718 (12)	0.045 (5)*	
C1	1.0046 (2)	0.93888 (19)	0.38352 (14)	0.0601 (6)	
H1A	0.9678	0.9089	0.4416	0.072*	
H1B	1.1011	0.9456	0.3857	0.072*	
C2	0.98194 (17)	0.84343 (17)	0.32713 (12)	0.0446 (4)	
H2A	1.0231	0.8702	0.2697	0.054*	
H2B	1.0239	0.7607	0.3506	0.054*	
C3	0.7737 (2)	0.9600 (2)	0.29057 (14)	0.0615 (6)	
H3A	0.6767	0.9551	0.2893	0.074*	
H3B	0.8100	0.9901	0.2324	0.074*	
C4	0.8019 (3)	1.0509 (2)	0.34844 (17)	0.0762 (7)	
H4A	0.7597	1.1345	0.3272	0.091*	
H4B	0.7620	1.0219	0.4059	0.091*	
C5	0.79770 (17)	0.73575 (17)	0.27916 (11)	0.0416 (4)	
H5	0.6984	0.7412	0.2832	0.050*	
C6	0.84088 (17)	0.60273 (18)	0.32558 (11)	0.0431 (4)	
C7	0.8263 (2)	0.5763 (2)	0.41468 (13)	0.0641 (6)	
H7	0.7950	0.6411	0.4449	0.077*	
C8	0.8572 (3)	0.4560 (2)	0.45923 (16)	0.0782 (8)	
H8	0.8476	0.4404	0.5191	0.094*	
C9	0.9018 (2)	0.3597 (2)	0.41592 (17)	0.0721 (7)	
H9	0.9222	0.2781	0.4460	0.086*	
C10	0.9165 (2)	0.3834 (2)	0.32819 (16)	0.0655 (6)	
H10	0.9469	0.3178	0.2985	0.079*	
C11	0.88661 (19)	0.5043 (2)	0.28309 (13)	0.0542 (5)	
H11	0.8975	0.5193	0.2232	0.065*	
C12	0.75048 (18)	0.75042 (19)	0.12841 (12)	0.0470 (5)	
C13	0.80194 (18)	0.7748 (2)	0.03612 (12)	0.0501 (5)	
C14	0.8036 (3)	0.6824 (3)	−0.01283 (16)	0.0868 (8)	
H14	0.7759	0.6020	0.0117	0.104*	
C15	0.8471 (3)	0.7081 (4)	−0.1008 (2)	0.1097 (12)	
H15	0.8510	0.6444	−0.1342	0.132*	
C16	0.8837 (3)	0.8282 (5)	−0.13637 (18)	0.1034 (11)	
H16	0.9098	0.8467	−0.1948	0.124*	
C17	0.8821 (3)	0.9187 (4)	−0.0879 (2)	0.1071 (11)	
H17	0.9080	0.9996	−0.1126	0.128*	
C18	0.8423 (3)	0.8927 (3)	−0.00143 (16)	0.0831 (8)	
H18	0.8429	0.9562	0.0319	0.100*	
C19	0.3099 (2)	0.32634 (19)	0.35265 (15)	0.0621 (6)	
H19A	0.2771	0.3350	0.4109	0.074*	
H19B	0.2636	0.2585	0.3363	0.074*	
C20	0.27701 (19)	0.44881 (18)	0.29285 (13)	0.0512 (5)	
H20A	0.3042	0.4392	0.2339	0.061*	
H20B	0.1801	0.4700	0.2967	0.061*	
C21	0.49322 (17)	0.51686 (17)	0.31197 (13)	0.0474 (5)	
H21A	0.5411	0.5837	0.3284	0.057*	
H21B	0.5247	0.5080	0.2535	0.057*	
C22	0.5214 (2)	0.39299 (19)	0.37222 (14)	0.0591 (5)	
H22A	0.6180	0.3701	0.3695	0.071*	
H22B	0.4935	0.4039	0.4308	0.071*	
C23	0.30607 (16)	0.67541 (16)	0.26940 (10)	0.0355 (4)	
H23	0.2068	0.6819	0.2759	0.043*	
C24	0.35210 (16)	0.78231 (16)	0.30993 (11)	0.0369 (4)	
C25	0.36414 (19)	0.90344 (17)	0.26293 (12)	0.0479 (5)	
H25	0.3521	0.9183	0.2041	0.057*	
C26	0.3938 (2)	1.00258 (19)	0.30196 (14)	0.0574 (5)	
H26	0.4007	1.0837	0.2694	0.069*	
C27	0.4132 (2)	0.98284 (19)	0.38818 (15)	0.0588 (6)	
H27	0.4330	1.0501	0.4143	0.071*	
C28	0.4031 (2)	0.8632 (2)	0.43562 (14)	0.0618 (6)	
H28	0.4173	0.8489	0.4941	0.074*	
C29	0.3720 (2)	0.76369 (18)	0.39714 (12)	0.0509 (5)	
H29	0.3643	0.6830	0.4302	0.061*	
C30	0.24685 (18)	0.70289 (18)	0.11932 (11)	0.0440 (4)	
C31	0.29382 (18)	0.7137 (2)	0.02652 (12)	0.0536 (5)	
C32	0.2052 (2)	0.7115 (3)	−0.03215 (14)	0.0763 (7)	
H32	0.1141	0.7053	−0.0143	0.092*	
C33	0.2436 (3)	0.7181 (3)	−0.11813 (15)	0.0907 (9)	
H33	0.1813	0.7438	−0.1599	0.109*	
C34	0.355 (2)	0.6909 (19)	−0.1370 (12)	0.088 (3)	0.337 (5)
H34	0.3805	0.6916	−0.1950	0.105*	0.337 (5)
C35	0.4530 (12)	0.6576 (19)	−0.0792 (7)	0.151 (4)	0.337 (5)
H35	0.5428	0.6396	−0.0973	0.181*	0.337 (5)
C36	0.4148 (10)	0.6518 (15)	0.0041 (6)	0.112 (2)	0.337 (5)
H36	0.4689	0.6068	0.0463	0.134*	0.337 (5)
C34A	0.3751 (10)	0.7434 (8)	−0.1504 (5)	0.088 (3)	0.665 (5)
H34A	0.4027	0.7427	−0.2079	0.105*	0.665 (5)
C35A	0.4608 (5)	0.7689 (9)	−0.0943 (3)	0.151 (4)	0.665 (5)
H35A	0.5469	0.7938	−0.1145	0.181*	0.665 (5)
C36A	0.4223 (4)	0.7585 (7)	−0.0062 (3)	0.112 (2)	0.665 (5)
H36A	0.4809	0.7810	0.0310	0.134*	0.665 (5)
N1	0.83673 (14)	0.83410 (14)	0.32292 (9)	0.0431 (4)	
N2	0.83821 (16)	0.75434 (15)	0.18720 (9)	0.0433 (4)	
N3	0.34807 (13)	0.55046 (13)	0.31612 (9)	0.0369 (3)	
N4	0.34120 (15)	0.69325 (14)	0.17671 (9)	0.0397 (4)	
O1	0.94265 (16)	1.06078 (13)	0.35261 (10)	0.0716 (5)	
O2	0.63267 (14)	0.7272 (2)	0.14833 (10)	0.0927 (6)	
O3	0.45161 (15)	0.29292 (12)	0.35088 (9)	0.0622 (4)	
O4	0.12536 (13)	0.70222 (17)	0.14163 (9)	0.0694 (5)	

### Atomic displacement parameters (Å^2^)

**Table:**

	*U*^11^	*U*^22^	*U*^33^	*U*^12^	*U*^13^	*U*^23^
C1	0.0634 (13)	0.0525 (13)	0.0676 (15)	−0.0006 (10)	−0.0101 (11)	−0.0180 (11)
C2	0.0409 (10)	0.0458 (11)	0.0473 (11)	−0.0016 (8)	−0.0037 (8)	−0.0086 (9)
C3	0.0511 (12)	0.0625 (14)	0.0687 (15)	0.0135 (10)	−0.0069 (10)	−0.0117 (11)
C4	0.0826 (18)	0.0589 (15)	0.0865 (18)	0.0211 (12)	−0.0035 (13)	−0.0233 (13)
C5	0.0335 (9)	0.0555 (12)	0.0349 (10)	−0.0077 (8)	0.0019 (7)	−0.0050 (8)
C6	0.0382 (10)	0.0536 (12)	0.0379 (11)	−0.0148 (8)	−0.0018 (8)	−0.0046 (9)
C7	0.0928 (17)	0.0605 (14)	0.0413 (13)	−0.0270 (12)	−0.0030 (11)	−0.0057 (10)
C8	0.114 (2)	0.0689 (17)	0.0519 (14)	−0.0410 (14)	−0.0205 (13)	0.0112 (13)
C9	0.0731 (16)	0.0569 (15)	0.0826 (19)	−0.0201 (11)	−0.0242 (13)	0.0130 (13)
C10	0.0576 (14)	0.0537 (14)	0.0823 (18)	−0.0037 (10)	−0.0003 (11)	−0.0062 (12)
C11	0.0490 (12)	0.0623 (14)	0.0494 (12)	−0.0053 (9)	0.0026 (9)	−0.0069 (10)
C12	0.0378 (11)	0.0611 (12)	0.0406 (11)	−0.0096 (8)	−0.0041 (8)	−0.0011 (9)
C13	0.0401 (11)	0.0722 (14)	0.0375 (11)	−0.0085 (9)	−0.0079 (8)	−0.0037 (10)
C14	0.0871 (19)	0.118 (2)	0.0651 (17)	−0.0422 (16)	0.0116 (13)	−0.0345 (16)
C15	0.086 (2)	0.187 (4)	0.076 (2)	−0.031 (2)	−0.0014 (16)	−0.068 (2)
C16	0.078 (2)	0.182 (4)	0.0451 (16)	−0.008 (2)	−0.0085 (13)	−0.002 (2)
C17	0.120 (3)	0.116 (3)	0.065 (2)	−0.001 (2)	0.0143 (17)	0.0269 (19)
C18	0.104 (2)	0.0780 (18)	0.0583 (16)	−0.0079 (14)	0.0130 (14)	0.0062 (13)
C19	0.0649 (14)	0.0479 (12)	0.0772 (16)	−0.0198 (10)	0.0078 (11)	−0.0195 (11)
C20	0.0471 (11)	0.0500 (12)	0.0617 (13)	−0.0137 (8)	−0.0027 (9)	−0.0201 (10)
C21	0.0392 (10)	0.0445 (11)	0.0569 (12)	−0.0049 (8)	−0.0053 (8)	−0.0027 (9)
C22	0.0583 (13)	0.0475 (12)	0.0694 (14)	−0.0039 (9)	−0.0121 (10)	−0.0006 (10)
C23	0.0324 (9)	0.0438 (10)	0.0312 (10)	−0.0034 (7)	−0.0008 (7)	−0.0086 (8)
C24	0.0368 (9)	0.0380 (10)	0.0355 (10)	−0.0018 (7)	−0.0026 (7)	−0.0059 (8)
C25	0.0562 (12)	0.0446 (11)	0.0407 (11)	−0.0013 (8)	−0.0034 (9)	−0.0021 (9)
C26	0.0654 (14)	0.0361 (11)	0.0685 (15)	−0.0055 (9)	−0.0002 (11)	−0.0042 (10)
C27	0.0681 (14)	0.0443 (12)	0.0702 (16)	−0.0089 (9)	−0.0114 (11)	−0.0213 (11)
C28	0.0883 (16)	0.0527 (13)	0.0496 (13)	−0.0093 (11)	−0.0188 (11)	−0.0147 (10)
C29	0.0737 (14)	0.0408 (11)	0.0395 (11)	−0.0107 (9)	−0.0117 (9)	−0.0043 (9)
C30	0.0349 (10)	0.0597 (12)	0.0387 (11)	−0.0010 (8)	−0.0053 (8)	−0.0112 (9)
C31	0.0371 (11)	0.0869 (15)	0.0369 (11)	0.0045 (9)	−0.0049 (8)	−0.0139 (10)
C32	0.0602 (14)	0.129 (2)	0.0433 (14)	−0.0190 (13)	−0.0053 (11)	−0.0173 (13)
C33	0.092 (2)	0.142 (3)	0.0416 (15)	−0.0070 (18)	−0.0130 (13)	−0.0222 (15)
C34	0.075 (4)	0.148 (8)	0.030 (3)	0.011 (5)	0.008 (2)	−0.001 (4)
C35	0.056 (2)	0.341 (12)	0.035 (2)	−0.024 (5)	0.0031 (17)	0.025 (5)
C36	0.0525 (19)	0.244 (8)	0.0336 (18)	−0.045 (4)	−0.0036 (13)	0.004 (4)
C34A	0.075 (4)	0.148 (8)	0.030 (3)	0.011 (5)	0.008 (2)	−0.001 (4)
C35A	0.056 (2)	0.341 (12)	0.035 (2)	−0.024 (5)	0.0031 (17)	0.025 (5)
C36A	0.0525 (19)	0.244 (8)	0.0336 (18)	−0.045 (4)	−0.0036 (13)	0.004 (4)
N1	0.0375 (8)	0.0483 (9)	0.0431 (9)	−0.0001 (6)	0.0002 (6)	−0.0089 (7)
N2	0.0311 (9)	0.0633 (11)	0.0344 (9)	−0.0083 (7)	0.0000 (7)	−0.0039 (7)
N3	0.0358 (8)	0.0381 (8)	0.0388 (8)	−0.0082 (6)	−0.0018 (6)	−0.0098 (6)
N4	0.0307 (8)	0.0575 (10)	0.0313 (8)	−0.0034 (7)	−0.0017 (6)	−0.0088 (7)
O1	0.0799 (11)	0.0464 (9)	0.0908 (12)	0.0007 (7)	−0.0088 (9)	−0.0186 (8)
O2	0.0414 (9)	0.184 (2)	0.0517 (10)	−0.0374 (10)	−0.0043 (7)	−0.0024 (11)
O3	0.0682 (10)	0.0396 (8)	0.0784 (11)	−0.0026 (6)	−0.0007 (8)	−0.0117 (7)
O4	0.0316 (8)	0.1312 (14)	0.0459 (8)	−0.0037 (7)	−0.0032 (6)	−0.0174 (9)

### Geometric parameters (Å, °)

**Table:**

C1—O1	1.416 (2)	C20—H20A	0.9700
C1—C2	1.508 (3)	C20—H20B	0.9700
C1—H1A	0.9700	C21—N3	1.455 (2)
C1—H1B	0.9700	C21—C22	1.506 (3)
C2—N1	1.459 (2)	C21—H21A	0.9700
C2—H2A	0.9700	C21—H21B	0.9700
C2—H2B	0.9700	C22—O3	1.420 (2)
C3—N1	1.460 (2)	C22—H22A	0.9700
C3—C4	1.502 (3)	C22—H22B	0.9700
C3—H3A	0.9700	C23—N3	1.452 (2)
C3—H3B	0.9700	C23—N4	1.461 (2)
C4—O1	1.416 (3)	C23—C24	1.520 (2)
C4—H4A	0.9700	C23—H23	0.9800
C4—H4B	0.9700	C24—C25	1.381 (2)
C5—N1	1.448 (2)	C24—C29	1.385 (2)
C5—N2	1.460 (2)	C25—C26	1.378 (3)
C5—C6	1.524 (3)	C25—H25	0.9300
C5—H5	0.9800	C26—C27	1.368 (3)
C6—C11	1.375 (3)	C26—H26	0.9300
C6—C7	1.385 (3)	C27—C28	1.368 (3)
C7—C8	1.374 (3)	C27—H27	0.9300
C7—H7	0.9300	C28—C29	1.381 (3)
C8—C9	1.362 (4)	C28—H28	0.9300
C8—H8	0.9300	C29—H29	0.9300
C9—C10	1.363 (3)	C30—O4	1.229 (2)
C9—H9	0.9300	C30—N4	1.339 (2)
C10—C11	1.381 (3)	C30—C31	1.491 (3)
C10—H10	0.9300	C31—C32	1.331 (3)
C11—H11	0.9300	C31—C36	1.376 (12)
C12—O2	1.219 (2)	C31—C36A	1.424 (5)
C12—N2	1.330 (2)	C32—C33	1.373 (3)
C12—C13	1.492 (3)	C32—H32	0.9300
C13—C14	1.357 (3)	C33—C34	1.15 (2)
C13—C18	1.369 (3)	C33—C34A	1.392 (9)
C14—C15	1.408 (4)	C33—H33	0.9300
C14—H14	0.9300	C34—C35	1.37 (2)
C15—C16	1.370 (5)	C34—H34	0.9300
C15—H15	0.9300	C35—C36	1.334 (16)
C16—C17	1.334 (4)	C35—H35	0.9300
C16—H16	0.9300	C36—H36	0.9300
C17—C18	1.379 (4)	C34A—C35A	1.350 (11)
C17—H17	0.9300	C34A—H34A	0.9300
C18—H18	0.9300	C35A—C36A	1.403 (7)
C19—O3	1.422 (2)	C35A—H35A	0.9300
C19—C20	1.500 (3)	C36A—H36A	0.9300
C19—H19A	0.9700	N2—H2	0.832 (19)
C19—H19B	0.9700	N4—H4	0.855 (19)
C20—N3	1.454 (2)		
			
O1—C1—C2	111.83 (17)	N3—C21—H21B	109.9
O1—C1—H1A	109.3	C22—C21—H21B	109.9
C2—C1—H1A	109.3	H21A—C21—H21B	108.3
O1—C1—H1B	109.3	O3—C22—C21	111.62 (16)
C2—C1—H1B	109.3	O3—C22—H22A	109.3
H1A—C1—H1B	107.9	C21—C22—H22A	109.3
N1—C2—C1	109.40 (15)	O3—C22—H22B	109.3
N1—C2—H2A	109.8	C21—C22—H22B	109.3
C1—C2—H2A	109.8	H22A—C22—H22B	108.0
N1—C2—H2B	109.8	N3—C23—N4	114.37 (13)
C1—C2—H2B	109.8	N3—C23—C24	111.98 (13)
H2A—C2—H2B	108.2	N4—C23—C24	112.06 (14)
N1—C3—C4	109.16 (17)	N3—C23—H23	105.9
N1—C3—H3A	109.8	N4—C23—H23	105.9
C4—C3—H3A	109.8	C24—C23—H23	105.9
N1—C3—H3B	109.8	C25—C24—C29	117.87 (16)
C4—C3—H3B	109.8	C25—C24—C23	121.25 (15)
H3A—C3—H3B	108.3	C29—C24—C23	120.65 (15)
O1—C4—C3	111.84 (17)	C26—C25—C24	120.91 (18)
O1—C4—H4A	109.2	C26—C25—H25	119.5
C3—C4—H4A	109.2	C24—C25—H25	119.5
O1—C4—H4B	109.2	C27—C26—C25	120.60 (19)
C3—C4—H4B	109.2	C27—C26—H26	119.7
H4A—C4—H4B	107.9	C25—C26—H26	119.7
N1—C5—N2	114.34 (14)	C26—C27—C28	119.35 (19)
N1—C5—C6	111.81 (14)	C26—C27—H27	120.3
N2—C5—C6	112.37 (15)	C28—C27—H27	120.3
N1—C5—H5	105.8	C27—C28—C29	120.38 (19)
N2—C5—H5	105.8	C27—C28—H28	119.8
C6—C5—H5	105.8	C29—C28—H28	119.8
C11—C6—C7	117.72 (19)	C28—C29—C24	120.88 (18)
C11—C6—C5	123.12 (16)	C28—C29—H29	119.6
C7—C6—C5	119.05 (17)	C24—C29—H29	119.6
C8—C7—C6	121.2 (2)	O4—C30—N4	121.70 (16)
C8—C7—H7	119.4	O4—C30—C31	120.55 (15)
C6—C7—H7	119.4	N4—C30—C31	117.75 (15)
C9—C8—C7	120.2 (2)	C32—C31—C36	109.8 (4)
C9—C8—H8	119.9	C32—C31—C36A	115.7 (2)
C7—C8—H8	119.9	C36—C31—C36A	47.6 (5)
C8—C9—C10	119.6 (2)	C32—C31—C30	120.02 (18)
C8—C9—H9	120.2	C36—C31—C30	119.3 (4)
C10—C9—H9	120.2	C36A—C31—C30	122.4 (2)
C9—C10—C11	120.5 (2)	C31—C32—C33	122.3 (2)
C9—C10—H10	119.8	C31—C32—H32	118.8
C11—C10—H10	119.8	C33—C32—H32	118.8
C6—C11—C10	120.8 (2)	C34—C33—C32	118.1 (10)
C6—C11—H11	119.6	C34—C33—C34A	25.3 (11)
C10—C11—H11	119.6	C32—C33—C34A	121.5 (4)
O2—C12—N2	121.92 (17)	C34—C33—H33	121.0
O2—C12—C13	120.76 (16)	C32—C33—H33	121.0
N2—C12—C13	117.31 (16)	C34A—C33—H33	111.7
C14—C13—C18	119.0 (2)	C33—C34—C35	123.9 (17)
C14—C13—C12	120.5 (2)	C33—C34—H34	118.1
C18—C13—C12	120.4 (2)	C35—C34—H34	118.1
C13—C14—C15	120.1 (3)	C36—C35—C34	117.6 (13)
C13—C14—H14	120.0	C36—C35—H35	121.2
C15—C14—H14	120.0	C34—C35—H35	121.2
C16—C15—C14	119.1 (3)	C35—C36—C31	118.8 (10)
C16—C15—H15	120.4	C35—C36—H36	120.6
C14—C15—H15	120.4	C31—C36—H36	120.6
C17—C16—C15	120.6 (3)	C35A—C34A—C33	117.0 (6)
C17—C16—H16	119.7	C35A—C34A—H34A	121.5
C15—C16—H16	119.7	C33—C34A—H34A	121.5
C16—C17—C18	120.3 (3)	C34A—C35A—C36A	121.2 (5)
C16—C17—H17	119.9	C34A—C35A—H35A	119.4
C18—C17—H17	119.9	C36A—C35A—H35A	119.4
C13—C18—C17	120.9 (3)	C35A—C36A—C31	120.1 (4)
C13—C18—H18	119.5	C35A—C36A—H36A	119.9
C17—C18—H18	119.5	C31—C36A—H36A	119.9
O3—C19—C20	111.83 (16)	C5—N1—C2	116.27 (13)
O3—C19—H19A	109.2	C5—N1—C3	113.02 (14)
C20—C19—H19A	109.2	C2—N1—C3	109.35 (15)
O3—C19—H19B	109.2	C12—N2—C5	121.87 (15)
C20—C19—H19B	109.2	C12—N2—H2	118.1 (13)
H19A—C19—H19B	107.9	C5—N2—H2	118.9 (13)
N3—C20—C19	109.53 (16)	C23—N3—C20	112.69 (13)
N3—C20—H20A	109.8	C23—N3—C21	115.67 (13)
C19—C20—H20A	109.8	C20—N3—C21	109.49 (14)
N3—C20—H20B	109.8	C30—N4—C23	121.95 (15)
C19—C20—H20B	109.8	C30—N4—H4	119.0 (12)
H20A—C20—H20B	108.2	C23—N4—H4	119.0 (12)
N3—C21—C22	109.04 (15)	C4—O1—C1	109.36 (17)
N3—C21—H21A	109.9	C22—O3—C19	109.97 (14)
C22—C21—H21A	109.9		
			
O1—C1—C2—N1	−58.3 (2)	C36A—C31—C32—C33	17.0 (5)
N1—C3—C4—O1	59.2 (2)	C30—C31—C32—C33	−178.5 (2)
N1—C5—C6—C11	−142.89 (17)	C31—C32—C33—C34	20.9 (12)
N2—C5—C6—C11	−12.8 (2)	C31—C32—C33—C34A	−8.1 (6)
N1—C5—C6—C7	41.1 (2)	C32—C33—C34—C35	−3(2)
N2—C5—C6—C7	171.26 (16)	C34A—C33—C34—C35	102 (4)
C11—C6—C7—C8	0.4 (3)	C33—C34—C35—C36	3(3)
C5—C6—C7—C8	176.60 (19)	C34—C35—C36—C31	−19 (2)
C6—C7—C8—C9	−0.7 (4)	C32—C31—C36—C35	32.9 (13)
C7—C8—C9—C10	0.4 (4)	C36A—C31—C36—C35	−74.3 (12)
C8—C9—C10—C11	0.1 (3)	C30—C31—C36—C35	177.1 (10)
C7—C6—C11—C10	0.1 (3)	C34—C33—C34A—C35A	−94 (3)
C5—C6—C11—C10	−175.94 (17)	C32—C33—C34A—C35A	−4.0 (10)
C9—C10—C11—C6	−0.3 (3)	C33—C34A—C35A—C36A	5.8 (12)
O2—C12—C13—C14	−63.2 (3)	C34A—C35A—C36A—C31	3.5 (11)
N2—C12—C13—C14	116.4 (2)	C32—C31—C36A—C35A	−14.8 (7)
O2—C12—C13—C18	113.7 (3)	C36—C31—C36A—C35A	79.2 (9)
N2—C12—C13—C18	−66.6 (3)	C30—C31—C36A—C35A	−178.9 (5)
C18—C13—C14—C15	0.4 (4)	N2—C5—N1—C2	−65.13 (19)
C12—C13—C14—C15	177.4 (2)	C6—C5—N1—C2	63.99 (19)
C13—C14—C15—C16	−1.9 (4)	N2—C5—N1—C3	62.6 (2)
C14—C15—C16—C17	2.0 (5)	C6—C5—N1—C3	−168.34 (15)
C15—C16—C17—C18	−0.6 (5)	C1—C2—N1—C5	−173.23 (15)
C14—C13—C18—C17	1.0 (4)	C1—C2—N1—C3	57.3 (2)
C12—C13—C18—C17	−176.0 (2)	C4—C3—N1—C5	171.06 (17)
C16—C17—C18—C13	−0.9 (4)	C4—C3—N1—C2	−57.7 (2)
O3—C19—C20—N3	−57.9 (2)	O2—C12—N2—C5	−2.3 (3)
N3—C21—C22—O3	58.8 (2)	C13—C12—N2—C5	178.09 (16)
N3—C23—C24—C25	158.18 (15)	N1—C5—N2—C12	−128.73 (18)
N4—C23—C24—C25	28.1 (2)	C6—C5—N2—C12	102.44 (19)
N3—C23—C24—C29	−27.4 (2)	N4—C23—N3—C20	−66.05 (18)
N4—C23—C24—C29	−157.45 (16)	C24—C23—N3—C20	165.10 (14)
C29—C24—C25—C26	−0.6 (3)	N4—C23—N3—C21	60.98 (18)
C23—C24—C25—C26	173.99 (17)	C24—C23—N3—C21	−67.87 (18)
C24—C25—C26—C27	0.6 (3)	C19—C20—N3—C23	−171.53 (15)
C25—C26—C27—C28	0.1 (3)	C19—C20—N3—C21	58.23 (19)
C26—C27—C28—C29	−0.8 (3)	C22—C21—N3—C23	172.89 (15)
C27—C28—C29—C24	0.8 (3)	C22—C21—N3—C20	−58.5 (2)
C25—C24—C29—C28	−0.1 (3)	O4—C30—N4—C23	2.9 (3)
C23—C24—C29—C28	−174.71 (18)	C31—C30—N4—C23	−176.67 (16)
O4—C30—C31—C32	−5.8 (3)	N3—C23—N4—C30	110.43 (18)
N4—C30—C31—C32	173.7 (2)	C24—C23—N4—C30	−120.76 (18)
O4—C30—C31—C36	−146.4 (7)	C3—C4—O1—C1	−58.9 (2)
N4—C30—C31—C36	33.2 (7)	C2—C1—O1—C4	58.3 (2)
O4—C30—C31—C36A	157.7 (4)	C21—C22—O3—C19	−57.6 (2)
N4—C30—C31—C36A	−22.7 (4)	C20—C19—O3—C22	57.2 (2)
C36—C31—C32—C33	−34.5 (7)		

### Hydrogen-bond geometry (Å, °)

**Table:**

*D*—H···*A*	*D*—H	H···*A*	*D*···*A*	*D*—H···*A*
N4—H4···O2	0.85 (2)	2.11 (2)	2.932 (2)	163.5 (17)
N2—H2···O4^i^	0.832 (19)	2.10 (2)	2.918 (2)	166.5 (17)

Symmetry codes: (i) *x*+1, *y*, *z*.
